# Advancing Towards Value-Based Integrated Care for Individuals and Populations

**DOI:** 10.5334/ijic.5450

**Published:** 2019-12-04

**Authors:** Roberto Nuño-Solinís

**Affiliations:** 1 Deusto Business School Health, University of Deusto, Bilbao, ES

**Keywords:** value-based health care, integrated care, outcomes measurement

Value-based health care (VBHC) is nowadays a global trend in healthcare management and policy [1]. Value is defined as the outcomes that matter to patients related to the costs required to achieve those outcomes [2]. VBHC is thus yet another strategy that promises to “fix” health care [3]. Although it is uncertain if this purpose will be achieved, it is clear that VBHC incorporates some very relevant elements that have been hitherto absent or neglected in the daily management of organizations and health systems.

To start with, a core aspect of the proposal must be highlighted here: VBHC calls for a measuring of health outcomes from a broad, plural perspective, and not merely from a health economics, primary care or other narrow scope. Not only that, but it also incorporates measuring health outcomes that matter to the patient as essential, i.e. patient-defined outcomes and patient-reported outcome measures [4]. This proposal and its instruments are not new, however it does constitute the definitive commitment to standardizing, systematizing and incorporating value-based thinking and functioning into the clinical and management routine. It involves breaking with the usual complacency of measuring activity, average stays, process indicators and resources, etc. and neglecting the true effects of healthcare interventions.

Thus, the widespread acceptance of VBHC would place us in scenarios involving organizational innovation, benchmarking and benchlearning, value-based purchasing, comparative effectiveness analysis and competition for value creation – the impact of which is not yet easy to foresee, but will lead to a disruption of the business model in health regardless of the type of health system prevailing in each country.

Following on from Porter and Teisberg’s proposals [1], other experts have also proposed their own theoretical models based on the underlying idea of value. For instance, there is the OECD’s Expert Panel on effective ways of investing in Health [5] defined in terms of value-based healthcare as a comprehensive concept founded on four value-pillars: appropriate care in order to achieve patients’ personal goals (personal value), achievement of best possible outcomes with available resources (technical value), fair resource distribution across all patient groups (allocative value) and contribution of healthcare to social participation and connectedness (societal value).

This definition can be useful at a macro level to ensure the financial sustainability of universal healthcare – a long-term strategy geared towards the reallocation of low-value and high-value care resources. However, it is not very actionable in clinical and management practice and, unlike Porter’s proposals, is not sufficiently nurtured by the advances made over the last few decades in Organization Theory, Strategy or Integrated Care.

From a care integration perspective, Porter’s proposals already incorporate a system integration vision [6] and anticipate three key changes: horizontal integration based on units of excellence that concentrate volume according to medical condition, moving non-acute care out of hospitals and multidisciplinary work into Integrated Practice Units (IPUs). The concept of IPUs was introduced in the book Redefining Health Care [1]. The term IPU was chosen to highlight the fact that whenever an organization is doing something complicated, it should organize itself around overall customer needs being met. IPUs are multidisciplinary teams organized around meeting the needs of groups of patients with a shared clinical condition.

Ultimately, at the core of VBHC there are already the seeds of a Value-Based Integrated Care vision (VBIC), but does it capture the theoretical and empirical advances of integrated care in recent decades? We believe there is a great path of conceptual and empirical progress between VBHC and integrated care that can eventually converge on a VBIC paradigm for both individuals and populations, as has been previously proposed by Valentijn and colleagues [7–8], although their concept of value is based on Berwick and colleagues’ Triple Aim model [9]. Therefore, Valentijn and Vrijhoef defined VBIC [8] as “patients’ achieved outcomes and experience of care in combination with the amount of money spent by providing accessible, comprehensive and coordinated services to a targeted population”.

Anyway, we can see that both proposals put forward by Porter and Berwick foster the implementation of integrated care delivery and new payment models that are key for transformation towards an era of value-based healthcare. There is also acknowledgement that successful implementation of care integration practices may provide the solutions needed to help improve patients’ care experiences and outcomes, and to minimize costs.

However, for there to be an evolution towards a VBIC paradigm, major theoretical challenges persist, such as:

– to effectively incorporate the population health vision into VBHC. This seems to be possible, although initial proposals put forward by VBHC have tended to be hospitalocentric, with a dominant medical vision and focused on improving results at the individual patient level.
– to align VBHC with the value of care in its broader sense, within an environment such as Europe in which most of healthcare and social expenditure is concentrated on a small number of people who are living with complex long-term conditions [10]. Bearing in mind the expected increase of a profile of patients with advanced age, frailty and pluripathology, etc. we need to rethink what value in care means (not only healthcare) and how to measure it in those vulnerable populations. As care delivery for these groups of people involves cross-sectoral and inter-professional collaborations, it seems to be challenging to identify and measure specific cycles of care as VBHC proposes.

At an implementation level, we can identify other methodological and operational challenges that require further research, such as the following:

– understanding the patient perspective is integral to delivering high-value, patient-centered care. However, not only patients, but also their informal caregivers and other relevant stakeholders, must be included in the development and establishment of outcome and experience measures, for example in the case of cognitive limitations or end-of-life care.
– similarly, there is a huge room for improvement in defining value and co-creating meaningful metrics with people and communities. This participatory approach must allow to represent social and other well-being outcomes that capture societal benefit of VBIC in its broader sense.
– multimorbidity has ceased to be the exception in the burden of disease of the population in high-income countries [11], and although standard sets of ICHOM and other initiatives consider comorbidities to be adjustment variables, a further step is still needed, especially in cases where the index diseases become multiple.
– outcome measures must be standardized in order to allow evaluation and benchmarking of specific conditions at an aggregate level, although they must also be sensitive enough to capture each patient’s individual needs and goals. Additionally, PROMs are based on subjectively-collected data with potential for unreliable measurement, and uncontrolled response bias that is not always fully understood [4]. Cultural variation and context specific variables must be taken into account as well.
– successful implementation of VBHC requires leadership, buy-in at clinical and managerial levels, as well as substantial resource investment in order to allow data collection (outcome measures and costs).

To sum up, we celebrate VBHC as an important advance, but also with caution, as it has the unintended potential to boost some fragmentation trends in health systems (hyperspecialization, disease focus, hospitalocentrism, etc.). The increasing number of people living with chronic conditions and population groups with complex health and social needs (palliative care, mental health, vulnerable groups, etc.) require not only healthcare, but also social and community support [12]. This fact cannot be overlooked and needs to be considered when reformulating new organizational models.

In an attempt to respond to this challenge, for many years now integrated care models have been emerging and advocating coordination between healthcare, social and community services, so as to remove the fragmented model of organization. It will be interesting to see how leading integrated care organizations adopt VBHC in their strategies and practices [13].

Finally, proposals such as VBIC that build on the fertile seed planted by integrated care literature are needed – proposals that incorporate a systemic vision and a focus on health/well-being rather than on disease, the role of communities, the value of care, all from a fair perspective. We think that the community of practitioners and researchers that revolve around IFIC may prove to be a key agent in building that exciting new model.
